# Long-term motor deficit in brain tumour surgery with preserved intra-operative motor-evoked potentials

**DOI:** 10.1093/braincomms/fcaa226

**Published:** 2021-01-23

**Authors:** Davide Giampiccolo, Cristiano Parisi, Pietro Meneghelli, Vincenzo Tramontano, Federica Basaldella, Marco Pasetto, Giampietro Pinna, Luigi Cattaneo, Francesco Sala

**Affiliations:** 1 Section of Neurosurgery, Department of Neurosciences, Biomedicine and Movement Sciences, University Hospital, Verona, Italy; 2 Division of Neurology and Intraoperative Neurophysiology Unit, University Hospital, Verona, Italy; 3 CIMEC—Center for Mind/Brain Sciences, University of Trento, Trento, Italy

**Keywords:** SMA-syndrome, intra-operative neurophysiological monitoring (IONM), motor-evoked potentials (MEPs), neuro-oncology, brain mapping

## Abstract

Muscle motor-evoked potentials are commonly monitored during brain tumour surgery in motor areas, as these are assumed to reflect the integrity of descending motor pathways, including the corticospinal tract. However, while the loss of muscle motor-evoked potentials at the end of surgery is associated with long-term motor deficits (muscle motor-evoked potential-related deficits), there is increasing evidence that motor deficit can occur despite no change in muscle motor-evoked potentials (muscle motor-evoked potential-unrelated deficits), particularly after surgery of non-primary regions involved in motor control. In this study, we aimed to investigate the incidence of muscle motor-evoked potential-unrelated deficits and to identify the associated brain regions. We retrospectively reviewed 125 consecutive patients who underwent surgery for peri-Rolandic lesions using intra-operative neurophysiological monitoring. Intraoperative changes in muscle motor-evoked potentials were correlated with motor outcome, assessed by the Medical Research Council scale. We performed voxel–lesion–symptom mapping to identify which resected regions were associated with short- and long-term muscle motor-evoked potential-associated motor deficits. Muscle motor-evoked potentials reductions significantly predicted long-term motor deficits. However, in more than half of the patients who experienced long-term deficits (12/22 patients), no muscle motor-evoked potential reduction was reported during surgery. Lesion analysis showed that muscle motor-evoked potential-related long-term motor deficits were associated with direct or ischaemic damage to the corticospinal tract, whereas muscle motor-evoked potential-unrelated deficits occurred when supplementary motor areas were resected in conjunction with dorsal premotor regions and the anterior cingulate. Our results indicate that long-term motor deficits unrelated to the corticospinal tract can occur more often than currently reported. As these deficits cannot be predicted by muscle motor-evoked potentials, a combination of awake and/or novel asleep techniques other than muscle motor-evoked potentials monitoring should be implemented.

## Graphical Abstract

**Graphical Abstract:**
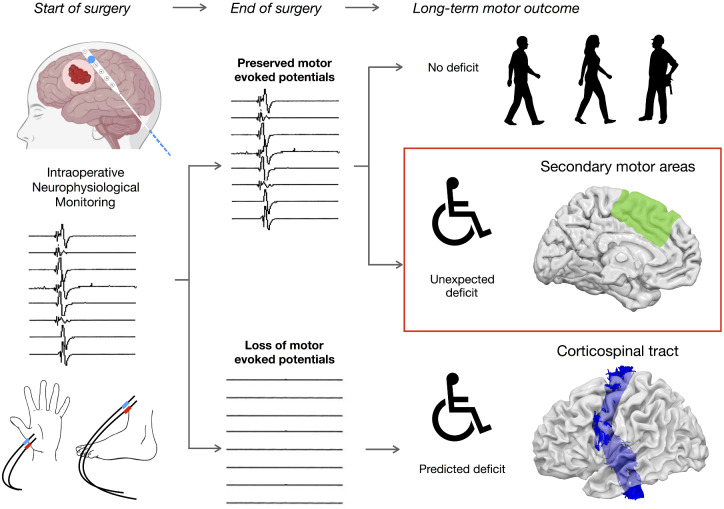

Abbreviated summary

In neurosurgery, disconnection of the corticospinal tract causes severe permanent deficits. Giampiccolo *et al.* retrospectively reviewed patients with lesions in motor areas and report that long-term motor deficits can occur following resection of secondary motor areas, even when motor-evoked potentials are preserved throughout surgery.

## Introduction

Prevention of motor dysfunction is a primary goal in brain surgery, since its impact on quality of life is profound (Osoba *et al*., 1996). Motor impairments often preclude further oncological treatments, which can impact on life expectancy (Weller *et al*., 2014). Pyramidal projections from the primary motor cortex must be preserved, as disconnection of the corticospinal tract (CST) is associated with post-operative hemiplegia (Penfield and Boldrey, 1937). Based on a train-of-five stimuli (To5) technique, developed by Taniguchi *et al.* (1993) to allow motor-evoked potential (MEP) monitoring under general anaesthesia, the use of a monopolar stimulation mapping technique has become a standard method for the identification of cortical and sub-cortical motor structures in both awake and asleep patients (Bello *et al.*, 2014). The To5 technique allows for both mapping and monitoring of the CST. Cortical and sub-cortical mapping can localize the CST online as the resection continues from the cortex into white matter. MEP monitoring tests the integrity of the entire corticofugal pathway, continuously throughout surgery, allowing for prediction as well as prevention of permanent motor deficits (Neuloh *et al.*, 2004).

A common assumption in brain surgery is that long-term motor deficits are ‘MEP-related’: a loss of MEPs at the end of surgery indicates that the patient will experience permanent hemiplegia, whereas no change in recorded MEPs from the start to end of the surgery indicates that motor function is preserved (Neuloh *et al.*, 2004). Yet, cases of transient (and even permanent) motor deficits occurring as a direct consequence of the surgery, not of post-operative events such as ischaemia or haemorrhage, but unrelated to MEP reduction have been increasingly described (Moser *et al.*, 2017), although reported for a low proportion of patients (3.5–11%) during supra-tentorial procedures (Neuloh and Schramm, 2009; Krieg *et al.*, 2012). Post-operative motor deficits without MEP reduction (from now: MEP-unrelated motor deficits) traditionally occur following lesion of the superior frontal gyrus (Zentner *et al.*, 1996; Seidel *et al.*, 2018). However, they have been described also for surgery of the middle frontal gyrus, the inferior frontal gyrus and even the pre-central gyrus (Moser *et al.*, 2017). Superior frontal gyrus-associated motor deficit is commonly termed as supplementary motor area (SMA) syndrome, a higher-order motor syndrome presenting with deficits in initiating and stopping movement attributed to resection of cortico-sub-cortical structures that gate primary motor output rather than that of corticospinal fibres (Nachev *et al.*, 2008). It should be noted that SMA syndrome is clinically characterized by MEP-unrelated hemi-akinesia: this condition of ‘behavioural hemiplegia’ despite preserved MEPs is not only an intra-operative phenomenon, but has been confirmed post-operatively using transcranial magnetic stimulation, thus excluding a motor deficit caused by CST damage (Zentner *et al.*, 1996; Sala *et al.*, 2000; Seidel *et al.*, 2018).

The value of MEPs in predicting and preventing motor deficit has been recently questioned. There is evidence that this monitoring technique cannot predict all possible motor deficits (Zentner *et al.*, 1996; Rossi *et al.*, 2018; Seidel *et al.*, 2018; Rech *et al.*, 2020) and might be blind to higher-order motor deficits. Therefore, it is critical to characterize the population of patients labelled as ‘false negatives’, understanding the incidence of this phenomenon, as it may represent a different patient population that cannot be protected by MEP monitoring. The aim of this study is to retrospectively describe the post-operative clinical profile (duration and severity of motor deficits) of a large cohort of patients with brain tumours involving motor areas, all operated on under MEP monitoring surveillance. We then correlated the clinical picture with intra-operative MEP results, considering any MEP amplitude drop of >50% as significant, a common criterion for MEP interpretation (Krieg *et al.*, 2012). Finally, we performed voxel–lesion–symptom mapping (VLSM) to identify brain regions associated with motor deficits, focussing on those unrelated to MEP reduction.

## Material and methods

### Patient demographics

Two hundred and fifty-two consecutive patients who underwent surgery for peri-Rolandic tumours using intra-operative neurophysiological monitoring (IONM) at the Institute of Neurosurgery in Verona from January 2012 to June 2016 were retrospectively reviewed. The inclusion criteria were: (i) a supra-tentorial brain tumour involving cortical and/or sub-cortical peri-Rolandic areas, (ii) surgical intervention using IONM with MEP monitoring for the upper limb, (iii) evaluation of motor status at four different time points (preoperatively, 2 days post-operatively, 5 days post-operatively and at a follow-up of minimum 3 months) and (iv) pre-operative motor performance higher than 4, evaluated using the Medical Research Council (MRC) scale. We decided to exclude all patients with clinically relevant pre-operative paresis (MRC < 4), since compromised pre-operative motor status may have biased motor outcome evaluation. Ischaemic or haemorrhagic post-surgical complications unrelated to MEP reduction were considered as exclusion criteria. Roughly half of the patients (125) satisfied the inclusion criteria. The study proposal is in accordance with ethical standards of the Declaration of Helsinki, and written consent was signed by all patients before surgical procedure.

### Surgical procedure and intra-operative neurophysiological monitoring

We applied a Total IntraVenous Anaesthesia (T.I.V.A.) protocol with a continuous infusion of Propofol (100–150 μg/kg/min) and Fentanyl (1 μg/kg/min), avoiding bolus. Halogenated anaesthetic agents were never used. Short-acting muscle relaxants were given only for intubation but not thereafter. The train-of-four technique was used to test the degree of muscle relaxation. Neurophysiological monitoring and mapping involved simultaneous acquisition of continuous electroencephalography—switched to electrocorticography as soon as the dura was opened—and electromyography (EMG) by means of the ISIS-IOM polygraph (Inomed Medizintechnik GmbH, Emmendingen, Germany).

Cortical and sub-cortical stimulations were performed using a monopolar probe (45 mm, angled 30°, Inomed Medizintechnik GmbH, Emmendingen, Germany) referenced to a scalp electrode at the Fz, delivering short train of five pulses (To5) (duration, 0.5 ms; ISI, 2–4 ms and repetition rate, 1 Hz). MEP and EMG were recorded via sub-dermal monopolar needle electrodes (Ambu^®^ Neuroline, Copenhagen, Denmark) in the upper (abductor pollicis brevis, extensor digitorum communis) and lower limb (quadriceps femoralis abductor hallucis and tibialis anterior). Once the dura was opened, MEP monitoring was continued using cortical rather than transcranial MEPs. Cortical MEP monitoring was performed using a 6- or 8-contact strip electrode (contact diameter, 2.5 mm; space, 10 mm; contact strips: 0.7 mm thin, 10 mm width, Inomed Medizintechnik GmbH, Emmendingen, Germany) placed over the pre-central gyrus.

### Assessment of intra-operative motor-evoked potential reduction

Continuous MEP recording was performed throughout the surgery. Opening (before any surgical resection) and closing (at the end of surgical resection and hemostasis) cortical MEP amplitudes were compared using a >50% drop criterion, as most commonly adopted (Krieg *et al.*, 2012). As persistent hemiplegia is described for MEP loss (Neuloh *et al.*, 2004), any reduction in MEPs (>90%) was also reported. Threshold stimulation intensity (mA) was defined as the lowest electric current allowing for a stable, reproducible, cortical MEPs (peak-to-peak amplitude, 100 μV) from the cortical motor hotspot in the pre-central gyrus, and recorded throughout the surgery. Dynamic MEP reduction of >50% during the operation which reversed to sub-threshold or complete MEP amplitude recovery was recorded. Deficits occurring in these patients were considered MEP related.

### Motor outcome evaluation

Motor performance involved separate evaluation of the upper limb (fingers, upper arm and lower arm) and the lower limb (toes, upper leg and lower leg) using the Medical Research Council (MRC) scale. Results for the lower limb are shown in the Supplementary Material. Motor strength scores for group muscles were averaged within a limb. Motor deficits were initially considered as reduction of pre-operative MRC score and classified as absent (no deficit) or present (deficit). Motor deficits were then sub-classified as mild (MRC reduction, ≤1), moderate (MRC reduction, >1 and ≤2) and severe (MRC reduction, >2). Patients were assessed for motor function pre-operatively, at 2 and 5 days post-operatively, and at a follow-up of at least 3 months. Patients with intact neurological function at 3 month follow-up were not further re-evaluated, since any worsening of motor status was considered as a recurrence of disease. Average of follow-up for patients with motor deficits was 25 months, with a MEP-unrelated motor deficits average follow-up of 40 months. This latter follow-up is longer because we wanted to re-assess as late as possible all patients who presented motor deficits not predicted by intra-operative MEPs, with the goal to define the severity and duration of the deficit.

### Neuropsychological evaluation of broader motor function

Living patients who suffered from long-lasting MEP-unrelated motor deficits were re-tested for broader neuropsychological deficits. Semi-quantitative assessment of motor function was made by the following set of standardized tests: handedness [Edinburgh Handedness test (Oldfield, 1971)]; grip force [hand-held dynamometer (Andrews *et al.*, 1996)]; fine motor skills [Finger tapping test (Hubel *et al.*, 2013)]; ideomotor apraxia (De Renzi *et al.*, 1980), visuomotor dexterity [9-hole Peg Test (Earhart *et al.*, 2011)] and motor impersistence (Joynt *et al.*, 1962). Finally, a short assessment of executive functions was performed by means of the Frontal Assessment Battery (Appollonio *et al.*, 2005).

### Voxel-based lesion symptom mapping

All included patients underwent post-operative CT (*n* = 55) or MRI (*n* = 70) scan within 48 h to evaluate post-surgical complications and the extent of resection*.* The surgical cavity was reconstructed using ITK-SNAP (Yushkevich *et al.*, 2006) and the individual brain anatomy with the related resection cavity was normalized to a template of 152 patients ( Montreal Neurological Institute) using enantiomorphic normalization from SPM12. We performed a voxel–lesion–symptom mapping analysis (Bates *et al.*, 2003) using NiiStat. All lesions were moved to the right hemisphere to increase numerosity and therefore statistical power. Proportion of resection in each region was entered into a general linear model to identify regions associated with motor deficits for the different time periods. The results of this analysis showed, as a *Z*-score, the statistical likelihood of resection of a given region predicting a decline in performance with respect to MEP alterations. Areas for decline in performance were sub-divided considering whether deficits were short term (post-operative reduction in MRC score that was resolved at follow-up) or long term (post-operative reduction in MRC score which persisted at follow-up). Finally, as both short-term and long-term MEP-unrelated deficits occurred for SMA resection, resection cavities for patient with short-term versus long-term MEP-unrelated deficits after resection of the superior frontal gyrus were compared.

### Sub-cortical white-matter anatomy

We evaluated the probability of disconnection of the CST using a white-matter atlas that is part of the Tractotron tool in BCB Toolkit software with a 50% probability threshold (Rojkova *et al.*, 2016).

### Statistical analysis

Statistical analysis was performed using STATA 15 (StataCorp LLC, USA). Normality of variable distribution was evaluated using a Kolmogorov–Smirnov test. A chi-square test or a Fisher’s exact test was used to evaluate homogeneity of groups. Independent *t*-test was used to determine significant means in two unrelated groups. Mann–Whitney rank test was used for non-parametric evaluation of independence of two groups. A multivariate analysis with correction for multiple comparison was performed for voxel-based analysis. The level of significance was *P* < 0.05.

### Data availability statement

The clinical data are available on reasonable request, in anonymized format, to the first or last author (D.G. or F.S.). Software used for data analysis included ITK-SNAP (www.itksnap.org), BCB Toolkit software (http://toolkit.bcblab.com), NiiStat (http://www.nitrc.org/projects/niistat) and SPM12 (https://www.fil.ion.ucl.ac.uk/spm/software/spm12/).

## Results

### Patients

One hundred and twenty-five patients (age, 49 ± 15 years; 49 F-76 M) fell within the defined inclusion criteria. In total, 64 patients (51.2%) had a right hemisphere lesion. Tumour localization was as follows: Rolandic (27 patients, 21.6%), pre-motor (34 patients, 27.2%), parietal (33 patients, 26.4%) and insular (31 patients, 24.8%). Demographic information on the patient group, extent of resection and tumour histology are summarized in Table 1. An overlay of overall patients’ resection cavities are described in Supplementary Fig. 1, as well as an illustrative case of a patient’s re-examination (Supplementary Illustrative case).

**Table 1:** Demographic and clinical information on patient group

Variables	Value	% of Patients
Patients	125	
Age	49 ± 15 years	
Sex		
Male	76	60.8
Female	49	39.2
Lesion side		
Left	61	48,8
Right	64	51.2
Tumour location		
Pre-motor	34	27.2
Rolandic	27	21.6
Parietal	33	26.4
Insular	31	24.8
Clinical presentation		
Confusion	5	4
Gait disturbances	2	1.6
Incidental	6	4.8
Language deficits	5	4
Limb paresthesia	1	0.8
Limb weakness	17	13.6
Recurrence on MRI	14	11.2
Seizure	75	60
Hospital length	11 ± 6 days	
Extent of resection (EoR)		
Total (100%)	75	60
Sub-total (90–99%)	47	37.6
Partial (70–99%)	3	2.4
Histology		
High-grade gliomas	88	70.4
Low-grade gliomas	14	11.2
Cavernoma	6	4.8
Ependymoma	2	1.6
Metastasis	6	4.8
Atypical meningioma	6	4.8
Lymphoma	2	1.6
DNET	1	0.8

## Motor outcome

### Upper limb

**Figure 1:**
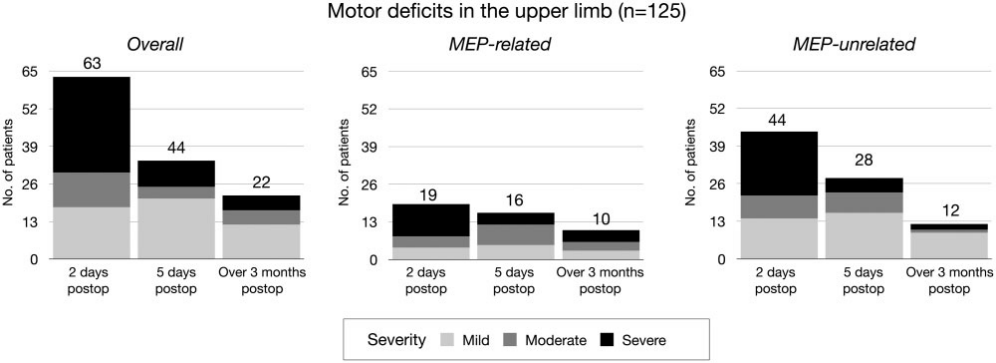
**Motor outcome in relationship with MEP reduction for upper and lower limb**. Upper limb: Motor deficits are shown in the left column and further sub-divided according to MEP variation into MEP-related (MEP drop >50% amplitude) and MEP-unrelated (motor deficit with no significant MEP-reduction). The bar charts show that MEP-unrelated deficits accounted for majority of deficits in the post-operative phase and half of the motor deficits at follow-up. MEP-unrelated motor deficits were fewer but more severe, normally long-lasting. *Severity of motor deficits = Mild (MRC, ≤1), Moderate (MRC, >1 and ≤2) and Severe (MRC, >2)*.

Motor outcome in the upper limb and its relationship with MEP reduction at different time points are shown and summarized in Fig. 1 and in Table 2.

**Table 2:** MEP reduction and motor outcome in the upper limb

Variable	No. of patients	% of patients
Upper limb MEP reduction >50%		
Yes	20	16
No	105	84
Preoperative MRC score in the upper limb		
5	92	73.6
4.5	3	2.4
4	30	24
MRC variation at 2 days after surgery in the upper limb		
None	61	48.8
≤1 MRC reduction	18	14.4
MRC reduction, >1 and ≤2	12	9.6
MRC reduction, >2	33	26.4
MRC increase compared to pre-operative status	1	0.8
MRC variation at 5 days after surgery in the upper limb		
None	73	58.4
MRC reduction, ≤1	21	16.8
MRC reduction, >1 and ≤2	14	11.2
MRC reduction, >2	9	7.2
MRC increase compared to pre-operative status	8	6.4
MRC variation at follow-up after surgery in the upper limb		
None	90	72
MRC reduction, ≤1	12	9.6
MRC reduction, >1 and ≤2	4	3.2
MRC reduction, >2	6	4.8
MRC increase compared to pre-operative status	13	10.4
Increase in current intensity (mA)		
Yes	38	30.4
No	87	69.6

#### Upper limb motor outcome

Twenty-six patients (21%) showed MEP reduction (with MEP reduction >90% in 10 patients and dynamic MEP reduction in 6 patients), whereas 99 (79%) showed no significant MEP alteration.

Post-operatively, half of the patients (63 patients; 50.4%) suffered from motor deficits (33 severe, 12 moderate, 18 mild), whereas the other half (62; 49.6%) had preserved motor function. Evaluation after 5 days showed a trend towards recovery: a third of patients (44 patients; 35.2%; 9 severe, 14 moderate, 21 mild) presented motor deficits with 81, showing no new motor deficit. At follow-up, long-term motor deficits occurred in almost a fifth of patients (22, 17.6%; 5 severe, 5 moderate, 12 mild) with preserved motor ability in 103 patients (82.4%).

#### Motor deficits and MEP reduction in the upper limb

Of 63 patients showing early post-operative motor deficits, more than two-thirds occurred without MEP reduction (44/63, 70%; 22 severe, 8 moderate, 14 mild) with 19/63 patients (30%, 11 severe, 4 moderate, 4 mild) associated with MEP reduction (including dynamic MEP reduction, five patients). After 5 days, the number of patients with motor deficits and no MEP reduction decreased sharply 28/44 patients (5 severe, 7 moderate, 16 mild), whereas the number of patients suffering from motor deficits with MEP drop remained similar (16/44; four severe, seven moderate, five mild and three patients with dynamic MEP reduction). At follow-up, around half of the patients with long-term motor deficits were those with no significant MEP reduction [12/22 (two severe, one moderate and nine mild)]. The remaining 10 patients (four severe, three moderate and three mild) had shown MEP reduction at the end of surgery. MEP reduction was significantly associated with long-term motor deficit (*P* < 0.001). Notably, 7 out of 10 patients with long-term deficit had an MEP reduction of >90%.

#### Motor deficits and current intensity

In 38/125 patients, a higher current intensity was required to obtain a stable MEP, with 19 of those requiring an increase of 5 mA or higher. Increased current intensity was significantly associated with moderate to severe motor deficits [*t*(123) = 4.12 *P* < 0.0001]. This was associated to MEP-related deficits [*t*(111)=4.63 *P* < 0.0001]. Conversely, it was not associated with MEP-unrelated deficits [*t*(113)=1.85 *P* = 0.66].

#### Motor deficits and extent of resection

We performed a Pearson’s chi-square test to investigate whether extent of resection was associated with a worse neurological outcome, which was non-significant in the short-term cohort [*χ*^2^(2, *N* = 125) = 3.11 *P* = 0.21] as well as in the long-term cohort [*χ*^2^(2, *N* = 125)= 0.46 *P* = 0.54]. This was confirmed also for short-term [*χ*^2^(2, *N* = 115) = 0.46 *P* = 0.79] as well as long-term [*χ*^2^(2, *N* = 115) = 1.68 *P* = 0.43] MEP-unrelated cases in isolation. Similarly, neurological outcome was not associated with patient’s age [overall: *t*(123)=0.7 *P* = 0.46; MEP-unrelated: *t*(113) = −0.91 *P* = 0.36], tumour type [overall: *χ*^2^(6, *N* = 125)=2.5 *P* = 0.86; MEP-unrelated: *χ*^2^(6, *N* = 115)=2.77 *P* = 0.90] or tumour grading [overall: *χ*^2^(4, *N* = 125) = 4.60 *P* = 0.33; MEP-unrelated: *χ*^2^(4, *N* = 115) = 1.29 *P* = 0.86].

### Neuropsychological evaluation in patients suffering from long-lasting motor-evoked potential-unrelated motor deficits for the upper limb

Five patients out of 12 suffering from long-lasting MEP-unrelated deficits in the upper limb were re-evaluated. Results are summarized in Table 3. All patients were right-handers*.* Overall, all patients showed deficits in hand dexterity and executive functions, with grip force and visuo-motor dexterity also impaired when tested. In contrast, only one patient scored below normality when tested for ideomotor apraxia.

**Table 3:** Neuropsychological evaluation in the long-term MEP-unrelated motor deficits

Patient no.	Age (years)	Education (years)	FTT aff side (no tap)	FTT not aff side (no tap)	Dyn aff side (kg)	Dyn not aff side (kg)	Peg board aff side (s)	Peg board not aff side (s)	FAB aff side (*x*/18)	FAB not aff side (*x*/18)	De Renzi test (*x*/72)	Benton test (*x*/8)
2	56	8	40^**^	38^**^	na	na	na	na	11^**^	9^**^	56^*^	na
3	48	8	12^**^	37^**^	na	na	na	na	12^**^	13^**^	68	na
21	52	18	23^**^	50	na	na	na	na	11^**^	15	72	na
43	70	5	25^**^	53	16^**^	32	191^**^	22	8^**^	8^**^	64	1^**^
88	46	13	41^*^	59	24^**^	45	176^**^	17	15	16	72	8

*Abbreviations*: FTT, finger tapping test; Dyn, dynamometer; FAB, frontal assessment battery; na, not available.

* *Scores below normality*

** *Scores significantly below normality*.

## Voxel–lesion–symptom mapping and sub-cortical analysis

### Upper limb

**Figure 2:**
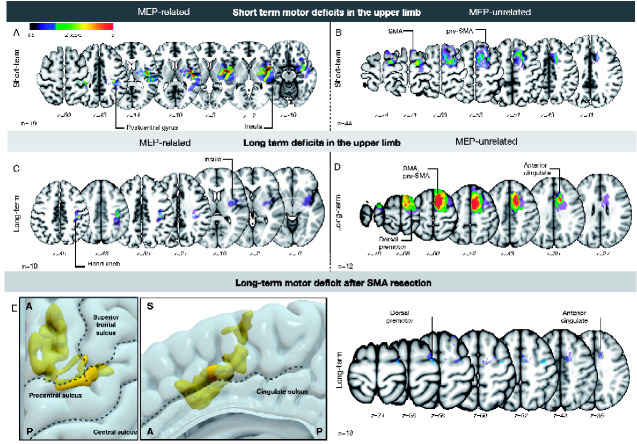
**VLSM analysis for MEP reduction and upper limb deficits. Short-term**: (**A**) MEP-related short-term deficits occurred mainly for insular resections, whereas (**B**) MEP-unrelated short-term motor deficits were associated with damage of the pre-SMA and SMA. *Long-term*: (**C**) MEP-related long-term deficits in the upper limb occurred after lesioning of the white-matter deep within the hand knob as well as after insular resection, suggesting damage to the corticospinal tract. (**D**) On the other hand, MEP-unrelated deficits occurred when re-secting the pre-SMA and SMA combined with the dorsal pre-motor cortex and the anterior cingulate cortex*. Long-term deficits for SMA resection*: (**E**) re-section of the SMA caused both short-term and long-term MEP-unrelated deficits in our cohort of patients. When contrasting these two cohorts of patients, long-term MEP-unrelated deficits occurred when SMA and pre-SMA as well as the dorsal pre-motor to the anterior cingulate cortex were re-sected. In contrast, no other area was associated with short-term deficits (data not shown).

Brain regions associated with upper limb deficits and MEP reduction are shown in Fig. 2.

#### Motor-evoked potential reduction in the upper limb

We performed a VLSM analysis to evaluate which regions corresponded with MEP reduction in the upper limb. MEP reduction occurred for resection of the insula, the corona radiata and the hand knob in the pre-central gyrus (*Z* = −2.87; *P* < 0.005; Supplementary Fig. 3a) and was associated with CST disconnection (*P* < 0.05).

#### Short-term motor deficits in the upper limb

Patients who suffered from post-operative deficits but recovered at follow-up were selected. Overall, short-term deficits corresponded with damage to the insula, the hand knob, the pre-SMA and SMA and a region deep within the superior parietal lobule (*Z* = −3.59; *P* < 0.001). Sub-cortical analysis for disconnection of the CST was not significant.

When dividing deficits according to MEP reduction, MEP-related short-term deficits occurred for re-section of the insula and the post-central gyrus (*Z* = −4.05; *P* < 0.001) (Fig. 2A), with MEP-unrelated motor deficits being associated with resection of the pre-SMA and SMA (*Z* = 2.77; *P* < 0.005) (Fig. 2B).

#### Long-term motor deficits in the upper limb

Re-section of the white matter within the hand knob, corona radiata, insular cortex, dorsal pre-motor cortex, pre-SMA, SMA and the anterior cingulate was associated with long-lasting motor deficits (*Z* = −3.1; *P* < 0.001). Sub-cortical analysis showed that long-term motor deficit was significantly related to disconnection of the CST (*P* < 0.03). We further sub-divided motor deficits according to MEP reduction. MEP-related long-term deficits corresponded with two regions: the insula and the sub-cortical white matter within the hand knob (*Z* = −2.51; *P* < 0.005) (Fig. 2C). On the other hand, MEP-unrelated motor deficits corresponded with the superior frontal gyrus, comprising the SMA and the pre-SMA, the dorsal pre-motor cortex and the anterior cingulate cortex (*Z* = 3.37; *P* < 0.001) (Fig. 2D).

### Comparison between regions associated with motor-evoked potential-unrelated short- and long-term deficits in the upper limb

As previously mentioned, both long- and short-term MEP-unrelated motor deficits occurred after re-section of the SMA and pre-SMA. We therefore compared re-sections of these two cohort of patients, investigating what lead to different long-term outcomes. Long-term MEP-unrelated motor deficit occurred when SMA and pre-SMA re-section was extended to the anterior cingulate cortex and the dorsal pre-motor cortex (*Z* = −2.85; *P* < 0.005) (Fig. 2E). Short-term deficits were associated with SMA and pre-SMA re-section in isolation. Since areas associated with long-term deficits were at re-section borders, their occurrence may have been related to a generally larger re-section rather than specific areas being co-re-sected. An independent *t*-test was performed to investigate whether the occurrence of long-term deficit was associated with larger resections, which was not significant [*t*(16) = 0.53 *P* = 0.59]. Moreover, we wanted to exclude that the difference between these two groups could be driven by a lesion to the CST with a sub-threshold (<50%) MEP reduction. A Mann–Whitney’s rank test showed no significant peri-operative MEP modification between these two groups (*Z* = −1.155 *P* = 0.24).

### Non-motor outcome

We frequently noticed language deficits occurring for re-section of the dominant SMA/preSMA, which occurred in five out of seven patients suffering from SMA-syndrome. Less frequently, non-dominant SMA/pre-SMA was accompanied by apathy/bradypsychia in 2/11 patients.

## Discussion

Post-operative neurological deficits negatively impact not only quality of life but also the survival of patients with brain gliomas (Weller *et al*., 2014; Rahman *et al.*, 2017). Therefore, the maintenance of an optimal onco-functional balance between maximizing re-section and minimizing morbidity is of paramount importance in neuro-oncology (Duffau and Mandonnet, 2013). Intra-operative neurophysiological cortical/sub-cortical motor mapping and monitoring of MEPs are considered to be the gold standard not merely to predict but possibly to prevent a post-operative hemiparesis or hemiplegia (Han *et al.*, 2018). In this study, we investigated the effectiveness of monitoring MEPs for surgery in motor areas. Our results confirm the role of MEP monitoring in the prediction and prevention of severe motor deficits, particularly when aiming to preserve the CST. However, our study shows that CST preservation alone may be insufficient to warrant motor function, as two-thirds of patients experienced short-term, and half of the patients long-term motor deficits without MEP reduction. From the perspective of the optimal functional–oncological balance, those with long-term motor deficits are of more concern: some degree of early, post-operative paresis despite preserved MEPs is reported and has heterogenous causes, ranging from damage to circuits for motor initiation to transitory ischaemia (Neuloh *et al.*, 2004; Seidel *et al.*, 2018). On the other hand, the possibility of long-term moderate or severe deficits that are not predicted by MEP monitoring is more important, especially for patients with high-grade glioma who may not have time for recovery. We therefore investigated whether this was linked to specific brain regions using VLSM, showing different clusters associated with long-term motor deficits. As expected, CST damage was associated with MEP loss during surgery, and consequent long-term motor deficits. Our results also indicate that re-section of the pre-SMA and SMA can be associated not only with short-term (SMA syndrome) but also with long-term motor deficits when pre-SMA and SMA resections were associated with damage to dorsal pre-motor and anterior cingulate cortices. Although preliminary, the results further suggest that either the so-called ‘SMA syndrome’ is not as transient and benign as reported previously (Zentner *et al.*, 1996; Tate *et al.*, 2011; Kim *et al.*, 2013) or alternatively, a long-lasting form also exists, occurring when damage is not limited to the SMA but extends to other frontal lobe areas, possibly damaging other motor control circuits. If so, a more sophisticated IONM approach may be needed, considering the tailoring of asleep versus awake surgery and/or the implementation of novel intra-operative motor testing.

### Motor-evoked potential-related motor deficits: motor-evoked potential reduction corresponds with damage to the corticospinal tract

It is well established that intra-operative MEP loss is associated with post-operative hemiplegia, which is reported for 58–100% of cases (Neuloh *et al.*, 2004; Szelényi *et al.*, 2010). This is assumed to be the result of either direct or ischaemic damage to the CST (Neuloh *et al.*, 2004). Our results support these previous reports, showing that MEP reduction had a high predictive value for long-term severe motor deficits. Moreover, long-term motor deficits were associated with white-matter re-section within the pre-central gyrus and the insular cortex. Clearly, direct disconnection occurs when damaging white matter of the pre-central gyrus: pyramidal tracts extend directly from this region and are involved in conveying motor output for different body parts (Penfield and Boldrey, 1937). On the other hand, trans-sylvian approaches to the insular cortex may cause secondary vascular ischaemia after insult to the anterior choroidal artery and M4 perforators supplying the corona radiata (Türe *et al.*, 2000; Neuloh *et al.*, 2007), which was confirmed in our cohort by MEP-dependent deficits when re-secting this area. Critically, sub-cortical analysis showed that both MEP reduction and long-term motor deficits were related to CST injury. Our data confirm the efficacy of MEP monitoring in evaluating descending pathways from the cortex to the muscles. They indicate that MEPs represent the neurophysiological marker for the integrity of the CST, as already suggested by other intra-operative (Zentner *et al.*, 1996) as well as extra-operative studies (Sala *et al.*, 2000; Seidel *et al.*, 2018).

### The role of motor-evoked potential-unrelated deficits in overall short-term deficits

Our results suggest that MEP reduction reflects disconnection of the CST. When performing MRC scoring, muscle strength is supposed to reflect activity in the corticospinal system, as MEPs and isometric strength are supposed to scale linearly (Townsend *et al.*, 2006). Accordingly, short-term MEP-related deficits occurred in vascular (insular cortex, post-central gyrus) or anatomical territories (internal capsule, pre-central gyrus) of the CST (Neuloh and Schramm, 2004; Neuloh *et al.*, 2007). However, voluntary movement involves a complex chain of events upstream from motor cortex activation, which could also result in muscle strength deficits. Any deficit in the brain’s capacity to collect and stably implement a motor command can result in a deficit in the MRC assessment which is not reflected by MEPs. This may correspond to two-thirds of short-term motor deficits in our cohort. These MEP-unrelated deficits differed to the others: they were initially severe but rapidly resolving, occurred for pre-motor and pre-frontal re-section and, critically, they were also often accompanied by other cognitive deficits. As a result, these data suggest that MEP preservation does not ensure that isometric strength will be maintained post-operatively, as damage to neural structures upstream from M1 also causes motor deficits, and therefore the surgeon should not rule out short-term deficits based on MEP preservation. To preserve motor function, higher-order motor circuits, also beyond the SMA, should be considered when aiming for the optimal onco-functional balance (Rossi *et al.*, 2018; Howells *et al*., 2018, 2020; Rech *et al.*, 2020).

### Motor-evoked potential-unrelated long-term motor deficits after supplementary motor area resection

MEP-unrelated motor deficits occurred after SMA and pre-SMA re-section which is consistent with SMA syndrome (Laplane *et al.*, 1977; Bannur and Rajshekhar, 2000) in which the patient suffers from *transient* post-operative akinesia, usually contralaterally. Reduced spontaneous motor activity occurs initially followed by complete long-term recovery despite impaired dexterity, normally between 7 and 10 days (Bannur and Rajshekhar, 2000), but sometimes up to 3 months (Fontaine *et al.*, 2002). In our series, long-term MEP-unrelated deficits occurred when SMA/pre-SMA re-section included the dorsal pre-motor and the anterior cingulate cortex: this was independent to the extent of re-section or even to sub-threshold MEP reductions. We speculate that this persistent form of SMA syndrome represents a motor control disorder, since the dorsal premotor cortex is involved in stimulus-based selection of hand movement (Cisek and Kalaska, 2005) and the anterior cingulate, alongside the SMA/pre-SMA, in internally driven generation of actions (Nachev *et al.*, 2005). Noticeably, our VLSM analysis did not show involvement of the corpus callosum. We want, however, to stress that a role for callosal re-section within the SMA/pre-SMA (Vergani *et al.*, 2014) must not be ruled out, especially when considering that long-lasting SMA syndrome has been reported for bilateral pre-motor tumorous lesions (Kofler *et al.*, 1999) and/or disturbance of the adjacent corpus callosum (Baker *et al.*, 2018).

To sum up, our preliminary data suggest that long-lasting motor deficits may exist for disconnection of anatomy involved in motor control. As shown by neuropsychological examination, these deficits may exceed isometric strength, impairing executive functions and dexterity. Compared to the classical *transient* SMA syndrome, this more severe form occurred after larger SMA ablation, including the anterior cingulate cortex and the pre-motor cortex, or the connection between these areas. Further studies in a larger cohort of patients should confirm these results, possibly providing its sub-cortical background and the clinical relevance.

### Long-term motor deficits and cortical mapping strategies

Our results confirm that MEP monitoring is highly effective in predicting motor deficits. However, they also suggest that there are conditions where preservation of the corticospinal alone is not sufficient to warrant motor function. We speculate that MEP monitoring (combined with sub-cortical mapping) may have changed the natural history of motor deficits for peri-Rolandic re-section in our cohort: damage to the CST progressively decreased causing a relative increase in non-corticospinal forms of paresis. However, our results question a surgery centred on CST preservation alone. It should be noted, however, that not every part of the premotor cortex is as important for preserving motor ability, as it has already been demonstrated using non-invasive (Bulubas *et al.*, 2016) as well as direct stimulation (Rossi *et al.*, 2018). Furthermore, it must be stressed that mild deficits may be a viable compromise to obtain the optimal onco-functional balance (Duffau and Mandonnet, 2013; Wijnenga *et al.*, 2018), as long-lasting deficits unrelated to intra-operative MEP reduction must, however, be considered rare. In different tumours, for example, low-grade gliomas compared to high-grade gliomas (HGGs), diverse life expectancies dictate the need to make tailored surgical choices between the benefit of a greater extent of resection, associated with increase of overall survival and the risk of neurological deficits. Thus, one might advise more conservative procedures to be applied to high-grade gliomas: the risk of even a temporary deficit may severely impair quality of life, which is even more relevant when life expectancy is lower. On the other hand, more aggressive ones could be performed for low-grade gliomas, considering that a deficit that is long-lasting, yet mild, might have an acceptable impact of quality of life when compared to enhanced survival. This holds also for stimulation protocols: asleep To5 may still be preferred to awake Penfield’s 50-Hz stimulation in mapping close to the CST or Rolandic tumours, since Penfield’s stimulation can incur up to 63% of false-negative mapping (Bello *et al.*, 2014). Asleep/awake procedures with a combined To5 and Penfield’s stimulation may be valuable for high-grade gliomas in the superior frontal gyrus, where post-operative MEP-unrelated deficits may pre-judice the benefit of a larger extent of resection, with the probe switched to To5 stimulation as the re-section approximates the CST (Bello *et al.*, 2014).

To conclude, the consolidated use of IONM in the form of MEP monitoring combined with sub-cortical dynamic mapping and tractography (Raabe *et al.*, 2014) has progressively allowed for safer surgery in terms of CST sparing (Raabe *et al.*, 2014). Yet, we observed a relative increase in non-corticospinal forms of paresis. Awake mapping of motor behaviour should be advocated for these dubious cases (Rossi *et al.*, 2018; Rech *et al.*, 2020) although novel methods in a fully asleep setting have recently been proposed (Cattaneo *et al.*, 2020). The combination of IONM strategies tailored to patient and tumour location should therefore be promoted.

## Conclusion

Our results indicate that two types of long-term motor deficits exist: one dependent on MEP reduction, the other independent of it. As an MEP drop predicts a permanent, severe motor deficits which is associated with disconnection of the CST, MEPs thus stand as its neurophysiological marker. However, our results also suggest that MEP-unrelated motor deficits may have an impact on patients’ outcome, particularly for cases in which SMA re-section occurs in conjunction with damage to the dorsal pre-motor and anterior cingulate cortex. Accordingly, awake as well as asleep techniques (Cattaneo *et al.*, 2020) other than MEP monitoring have to be adopted to avoid these deficits.

## Limitations

There are several limitations that need to be addressed. This is a retrospective study where we used the most common clinical scale for the evaluation of motor function, namely the MRC score. Second, it cannot be ruled out that MEP-unrelated deficits after SMA/pre-SMA re-section may arise from a mix of corticospinal and motor control deficits, as the lower limb M1 area extends more anteriorly than the upper limb motor area (Amunts and Zilles, 2015). Third, as much as we did not observe, in the post-operative MRI, evidence of venous infarction that may have explained the late onset of motor deficits, this possibility cannot be completely ruled out from a functional standpoint. Fourth, only patients with known MEP-unrelated deficits were prospectively re-evaluated for other deficits in motor behaviour, which is a limitation since patients with long-lasting deficits represent a minority of our patients. Accordingly, data showing that gross force deficits may represent epiphenomena of other higher-order motor syndromes are preliminary. In the future, prospective intra- and peri-operative examination of broader motor function, as performed in the illustrative case report, using tailored neuropsychological examination and kinematics may overcome this issue.

## Supplemental material

Supplementary material is available at *Brain Communications* online.

## Supplementary Material

fcaa226_Supplementary_Data

## Abbreviations

Biceps =: biceps brachii
CST =: corticospinal tract
EMG =: electromyography
ISI =: inter-stimulus interval
IONM =: intra-operative neurophysiological monitoring
Ment =: mentalis muscle
MEPs =: motor-evoked potentials
MRC =: Medical Research Council
OOM =: orbicularis oris
pre-SMA =: pre-supplementary motor area
SMA =: supplementary motor area
To5 =: train-of-five stimulation
VLSM =: voxel–lesion–symptom mapping
