# Supplementary material for: Long-term motor deficit in brain tumour surgery with preserved intra-operative motor-evoked potentials
Source: Brain Commun. 2021 Jan 23;3(1):fcaa226. doi: 10.1093/braincomms/fcaa226 (PMC7884605; doi:10.1093/braincomms/fcaa226)
Supplement: fcaa226_Supplementary_Data [file fcaa226_supplementary_data.docx]

**Supplementary material**

**Motor outcome in the lower limb**

Motor outcome in the lower limb and its relationship with MEP reduction at different time-points is shown in *Figure S2* and in *Table S1.*

*Lower limb motor outcome*

Of one hundred and twenty five patients, 64 had the lower limb additionally monitored, with 58 patients showing an MRC score 4 or higher. Of these, fourteen patients showed MEP reduction (4 of those with MEP reduction >90%) and 44 had no MEP reduction. Postoperatively, twenty-one patients suffered from lower limb motor deficits (10 severe, 5 moderate, 6 mild) and 37 had preserved motor function. The trend towards recovery after five days was less pronounced: motor deficits were still present in 16 of the 21 patients (4 severe, 5 moderate, 7 mild). At follow up, less than half of these patients recovered, with long-term motor deficits occurring in 13 patients (2 severe, 2 moderate, 9 mild).

*Motor deficits and MEP reduction in the lower limb*

Post-operatively, 13/21 patients showed motor deficits without MEP reduction (4 severe, 5 moderate, 4 mild) while 8/21 (6 severe, 3 mild) patients developed motor deficits with MEP reduction. Of those, two patients suffered from dynamic MEP reduction. After five days, patients with motor deficits and no MEP reduction were in 10/16 patients (2 severe, 2 moderate, 6 mild) with 6/16 patients (2 severe, 3 moderate, 1 mild) suffering from motor deficit and MEP drop over 50% (1 patients having dynamic MEP reduction). At follow-up, deficits without MEP reduction constitute the majority of motor deficits in the lower limb 10/13 (1 severe, 1 moderate, 8 mild), with 3/13 (1 severe, 1 moderate, 1 mild) patients showing deficits with MEP reduction (2 of them with an MEP reduction >90%).

*Comparison of MEP-unrelated motor deficits between lower limb and upper limb*

Lower limb deficits were fewer (13 compared to 23 in the upper limb), however, there were less patients with the lower limb monitored (58 compared to 125 upper limb patients). Accordingly, the incidence of deficits was similar: 13/58 (22.4%) for lower and 23/125 (18.4%) for upper limbs.

**Voxel-lesion-symptom mapping (VLSM) in the lower limb**

*MEP reduction in the lower limb*

MEP reduction occurred for resection of the corona radiata, the insula and dorsal precentral gyrus (z= -2.87; p<0.005) (Fig.S3b). VLSM analysis for short-term motor deficits in the lower limb was not significant and is not shown in Figure S3.

*Long-term motor deficits in the lower limb*

Regions emerging from the analysis were the precentral gyrus with the underlying white matter, the SMA and the pre-SMA (z= -3.37; p <0.001). MEP-related long-term deficits corresponded with the dorsal precentral gyrus and the underlying corona radiata (z= -2.37; p<0.01) (Fig. S3c). MEP-unrelated long-term deficits in isolation were not significant and are not shown.

**Comparison between long-term MEP-related deficits in the upper and the lower limb**

We compared whether the regions for motor deficits in the upper and the lower limb were somatotopically organised. This analysis was performed only for long-term MEP-related regions, since no other category reached significance for both limbs. As expected, the analysis showed that the region for upper limb MEP-related deficit was ventral to the one for the lower limb, converging subcortically.

**Supplementary Figures**

**
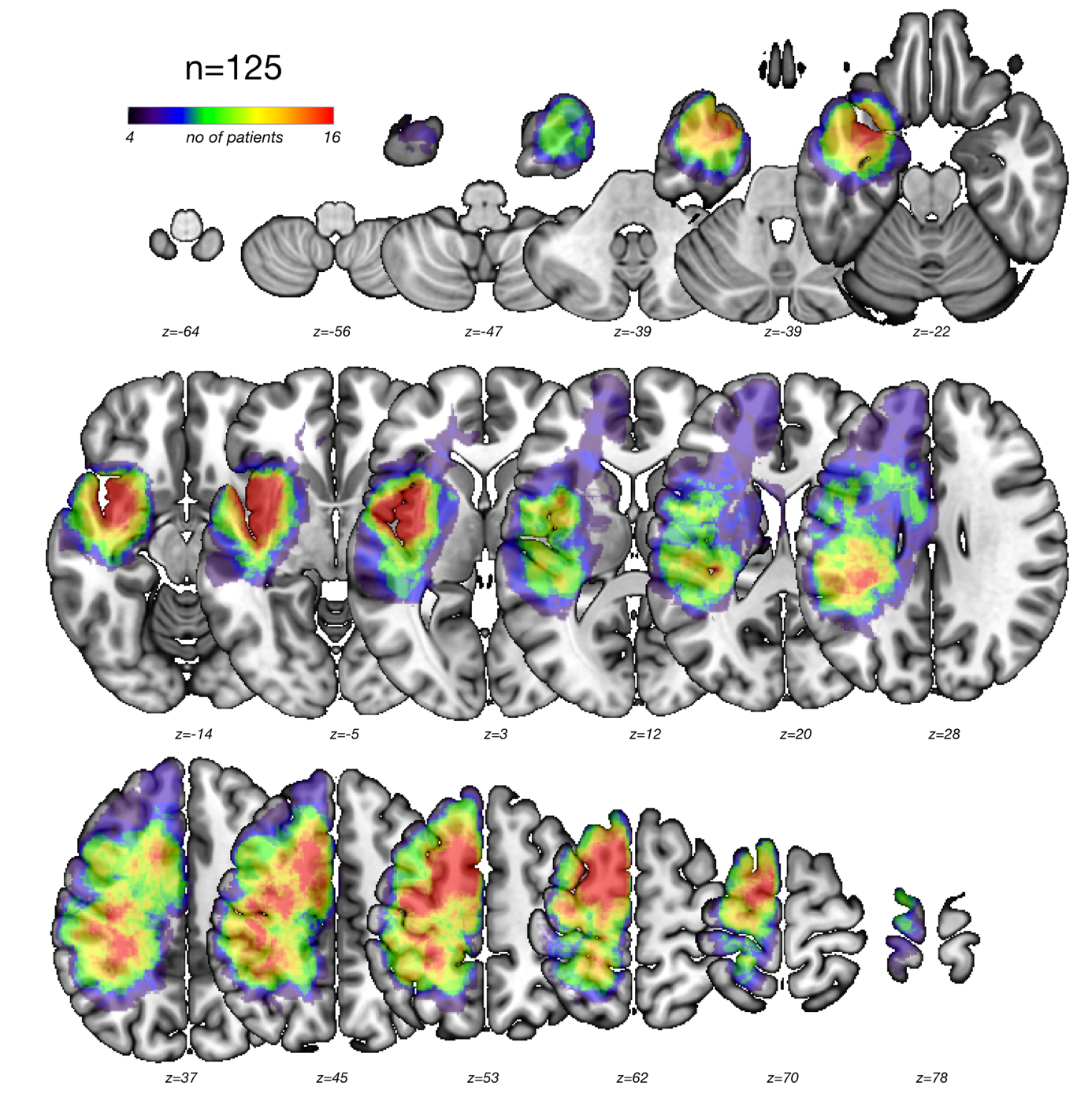
**

**Figure S1.** *Overlap of the resection cavities for the 125 patients undergoing IONM for tumours in cortico/subcortical peri-Rolandic areas*

**
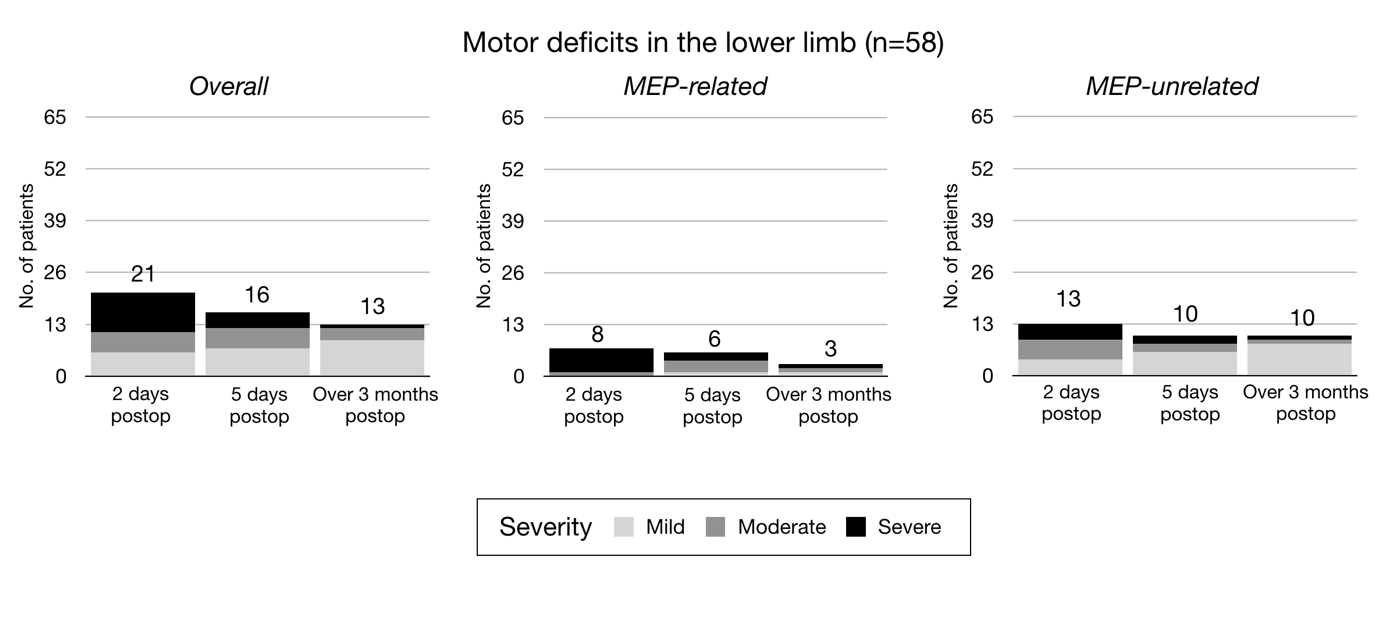
**

**Figure S2**. *Motor outcome in relationship with MEP reduction for upper and lower limb*. Motor deficits for the lower limb are shown in the left column and further subdivided according to MEP reduction into MEP-related (MEP drop > 50% amplitude) and MEP-unrelated (motor deficit with no significant MEP-reduction). The bar charts shows that MEP-unrelated deficits accounted for majority of deficits in both the postoperative phase and at follow-up, although were less severe than those MEP-related. *Severity of motor deficits= Mild (MRC ≤1), Moderate (MRC > 1 and ≤ 2), Severe (MRC >2)*

*
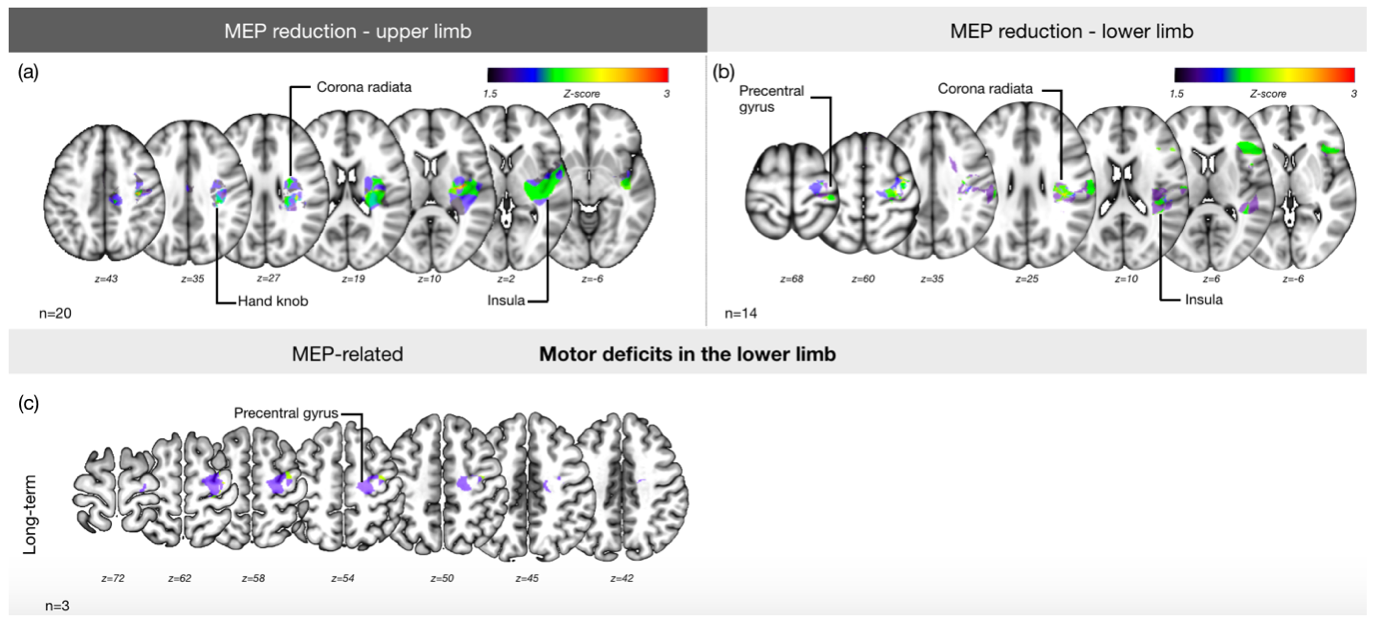
*

**Figure S3.** *VLSM analysis for MEP reduction in upper and lower limb and lower limb deficits.* MEP reduction in the upper limb (a) the analysis showed a region composed of the corona radiata, the hand knob and the insular cortex. In the lower limb MEP reduction occurred in (b) the dorsal precentral gyrus, the corona radiata and the insula. *Lower limb deficits:* (c) MEP-related long-term deficits occurred for damage to the dorsal precentral gyrus or the underlying corona radiata

**Supplementary Table**

*Table S1*: MEP reduction and motor outcome in the lower limb

| *Variable* | *No patients* | *% of patients* |
| --- | --- | --- |
| *Lower limb MEP reduction >50%* |  |  |
| *yes* | 14 | 24.1 |
| *no* | 44 | 75.9 |
| *Preoperative MRC score in the lower limb* |  |  |
| *5* | 49 | 84.5 |
| *4.5* | 1 | 1.7 |
| *4* | 8 | 13.8 |
| *MRC variation at 2-days after surgery in the lower limb* |  |  |
| *none* | 37 | 63.8 |
| *≤1 MRC reduction* | 6 | 10.3 |
| *MRC reduction > 1 and ≤2* | 5 | 8.6 |
| *MRC reduction >2* | 10 | 17.2 |
| *MRC increase compared to preoperative status* | 0 | 0 |
| *MRC variation at 5-days after surgery in the lower limb* |  |  |
| *none* | 39 | 67.2 |
| *≤1 MRC reduction* | 7 | 12.1 |
| *MRC reduction > 1 and ≤2* | 5 | 8.6 |
| *MRC reduction >2* | 4 | 6.9 |
| *MRC increase compared to preoperative status* | 3 | 5.2 |
| *MRC variation at follow-up after surgery in the lower limb* |  |  |
| *none* | 41 | 70.7 |
| *≤1 MRC reduction* | 9 | 15.5 |
| *MRC reduction > 1 and ≤2* | 2 | 3.4 |
| *MRC reduction >2* | 2 | 3.4 |
| *MRC increase compared to preoperative status* | 4 | 7 |
